# Purification and Structural Identification of Polysaccharides from Bamboo Shoots (*Dendrocalamus latiflorus*)

**DOI:** 10.3390/ijms160715560

**Published:** 2015-07-09

**Authors:** Jinsong Wu, Jiong Zheng, Xuejuan Xia, Jianquan Kan

**Affiliations:** 1 College of Food Science, Southwest University, Chongqing 400715, China; E-Mails: j2008173@swu.edu.cn (J.W.); zhengjiong248@163.com (J.Z.); xiaxuej1989@163.com (X.X.); 2 Chongqing Key Laboratory of Agricultural Product Processing, Southwest University, Chongqing 400715, China

**Keywords:** *Dendrocalamus latiflorus*, purification, structural identification

## Abstract

Three kinds of polysaccharides, namely, BSP1A, BSP2A, and BSP3B, were isolated from raw bamboo shoot (*Dendrocalamus latiflorus*) after purification and classification by DEAE cellulose-52 (ion-exchange chromatography) and Sephadex G-50. The molecular weights of BSP1A, BSP2A, and BSP3B were 10.2, 17.0 and 20.0 kDa, respectively, which were measured through GPC (gel performance chromtatography) methods. BSP1A contained arabinose, glucose, and galactose in a molar ratio of 1.0:40.6:8.7. BSP2A and BSP3B contained arabinose, xylose, glucose, and galactose in molar ratios of 6.6:1.0:5.2:10.4 and 8.5:1.0:5.1:11.1, respectively. The existence of the *O*-glycopeptide bond in BSP1A, BSP2A, and BSP3B was demonstrated by β-elimination reaction. FTIR spectra of the three polysaccharides showed that both BSP2A and BSP3B contained β-d-pyranose sugar rings. However, BSP1A exhibited both β-d-pyranose and α-d-pyranose sugar rings. Congo red test indicated that BSP1A and BSP2A displayed triple helix structures, but BSP3B did not. NMR spectroscopy revealed that BSP1A may exhibit a β-1,6-Glucan pyran type as the main link, and few 1,6-glycosidic galactose pyranose and arabinose bonds were connected; BSP2A mainly demonstrated →5)β-Ara(1→and→3)β-Gal(1→connection. Furthermore, BSP3B mainly presented →3)β-Glu(1→and→3)β-Gal(1→connection and may also contain few other glycosidic bonds.

## 1. Introduction

Bamboo shoot (*Dendrocalamus latiflorus*), which belongs to the giant grass of subfamily (Poaceae) of Bambusoideae plants, is rich in fiber and nutrients but low in fat and is widely distributed in China, Japan, and other southeast Asian countries [1,2]. Among the domestic shoot species bamboo shoot has the largest numbers of species, and this plant offers high nutritional value [3,4,5]. Moreover, bamboo shoot polysaccharide, extracted from *D. latiflorus* or other species with high research value, is involved in antioxidant activities.

To date, studies on bamboo shoot polysaccharides have mainly focused on the extraction process and provided little attention on purification and structure identification. β-d-Glucan and xyloglucan have been obtained from 4% and 24% potassium hydroxide extracts, respectively [6]. Three anticomplementary beta-glucans (BS-BGA, BS-BGB, and BS-BGC) have been isolated and characterized from bamboo shoots [7]. Bamboo shoot crude polysaccharides (BSCPs) extracted from the shoots of *Gigantochloa levis* yielded about 3.27% ± 0.18% on a dry basis and a very minute percentage of protein (0.02% ± 0.01%). These results indicated the potential to develop BSCPs as promising prebiotics [8]. Suzuki *et al.* reported that bamboo shoot hemicellulose polysaccharides extracted by alkaline hydrolysis act as anti-mouse tumor and anti-sarcoma 180 agents [9]. Therefore, to explore their biological activities the purification and preliminary identification of the structure of bamboo shoot polysaccharides are of significant research interest.

In the present study, three polysaccharides, namely, BSP1A, BSP2A, and BSP3B, were purified and isolated from raw bamboo shoot (*D. latiflorus*). BSP1A, BSP2A, and BSP3B were identified through GPC (gel performance chromtatography), monosaccharide composition analysis, glycopeptide bond connectivity analysis, infrared spectroscopy, Congo red test, and nuclear magnetic resonance (NMR) spectroscopy experiments. The results provided the first report on bamboo shoot (*D. latiflorus*) polysaccharides, which can facilitate further investigation about the structure–activity relationship and health food industry application of these polysaccharides.

## 2. Results and Discussion

### 2.1. Isolation and Purification of Bamboo Shoot Polysaccharides

**Figure 1:**
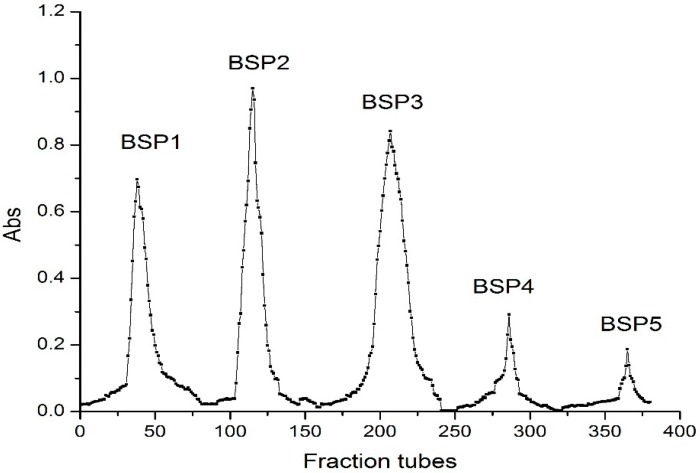
DEAE-cellulose-52 (ion-exchange chromatography) of BSP (Bamboo shoot polysaccharide). BSP1 eluted with water; BSP2 eluted with 0.05 M NaCl; BSP3 eluted with 0.1 M NaCl; BSP4 eluted with 0.2 M NaCl; BSP5 eluted with 0.5 M NaCl.

As was shown in Figure 1, fraction BSP1 comprised neutral polysaccharides as it was eluted with water. Other fractions (BSP2–BSP5) were acidic polysaccharides as they were eluted with increasing concentrations of NaCl from 0.05 to 0.50 M by using DEAE-Cellulose 52 [10,11]. The elution components were collected and calculated. The yields were 19.3%, 25.4%, 29.6%, 4.6%, and 2.2%. Given the very low yields of BSP4 and BSP5, BSP1, BSP2, and BSP3 were considered the main components of elution.

BSP1A, BSP2A, BSP3B were eluted using a gradient of water, 0.05 and 0.1 mol/L from each of BSP1, BSP2 and BSP3 respectively by Sephadex G-50 glucan gel elution. Afterward, BSP1A, BSP2A, and BSP3B were obtained. Purity identification results indicated that the three polysaccharides contained low levels of proteins and nucleic acids. Their purities were >95.3%; hence, they are pure polysaccharide substances and are used for further analysis.

### 2.2. Molecular Weight Determination

**Figure 2:**
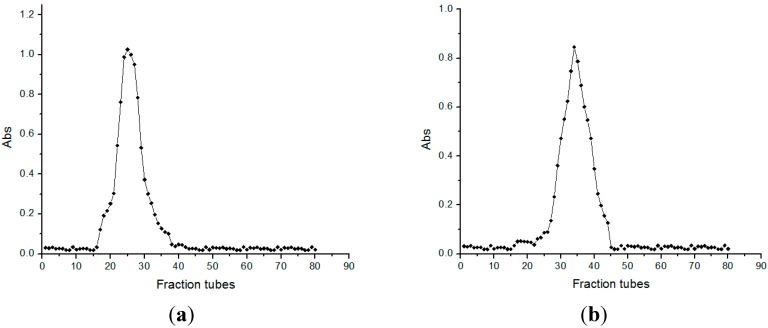

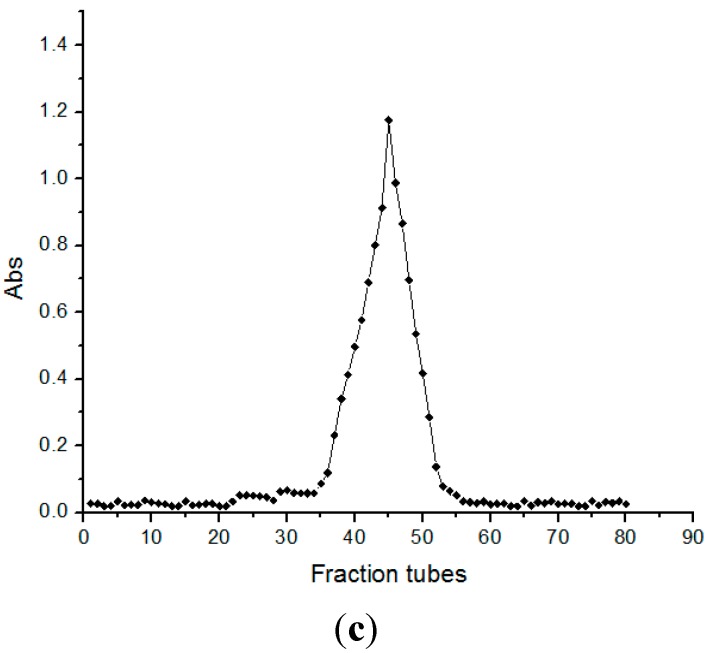
The result of molecular weight determination. (**a**) SephadexG-100 elution curve of BSP1A; (**b**) SephadexG-100 elution curve of BSP2A; (**c**) SephadexG-100 elution curve of BSP3B.

Ve and Vo were measured on the basis of the absorbance. Standard curve regression equation was calculated as follows: (Ve/Vo) = −1.5265 (lg*M*_W_) + 8.4538, *R*^2^ = 0.9987. The standard blue dextran V_0_ was 27 mL. As shown in Figure 2a–c, the absorbances of BSP1A, BSP2A, and BSP3B were >0.1 for tubes 17–37, 27–44, and 36–53. Tube elution volumes Ve were 63, 54, and 51 mL. On the basis of the Ve'/V_0_ values, the molecular weight of 10.2 kDa (BSP1A), 17.0 Da (BSP2A), and 20.0 kDa (BSP3B) were calculated.

### 2.3. Monosaccharide Composition

**Figure 3:**
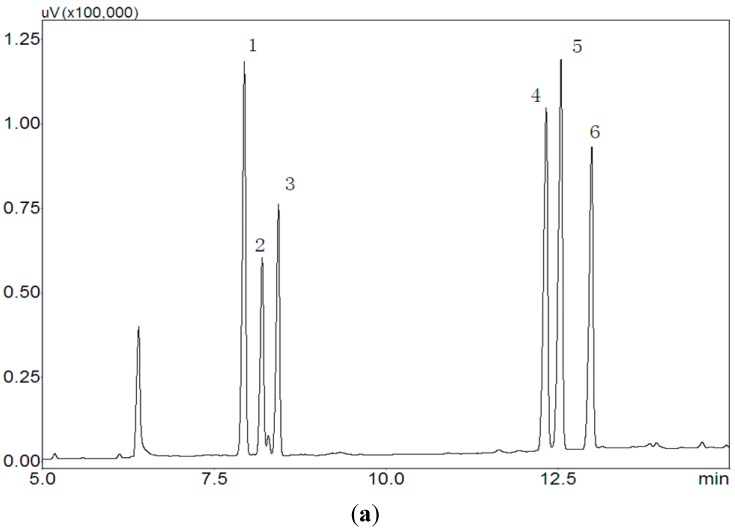

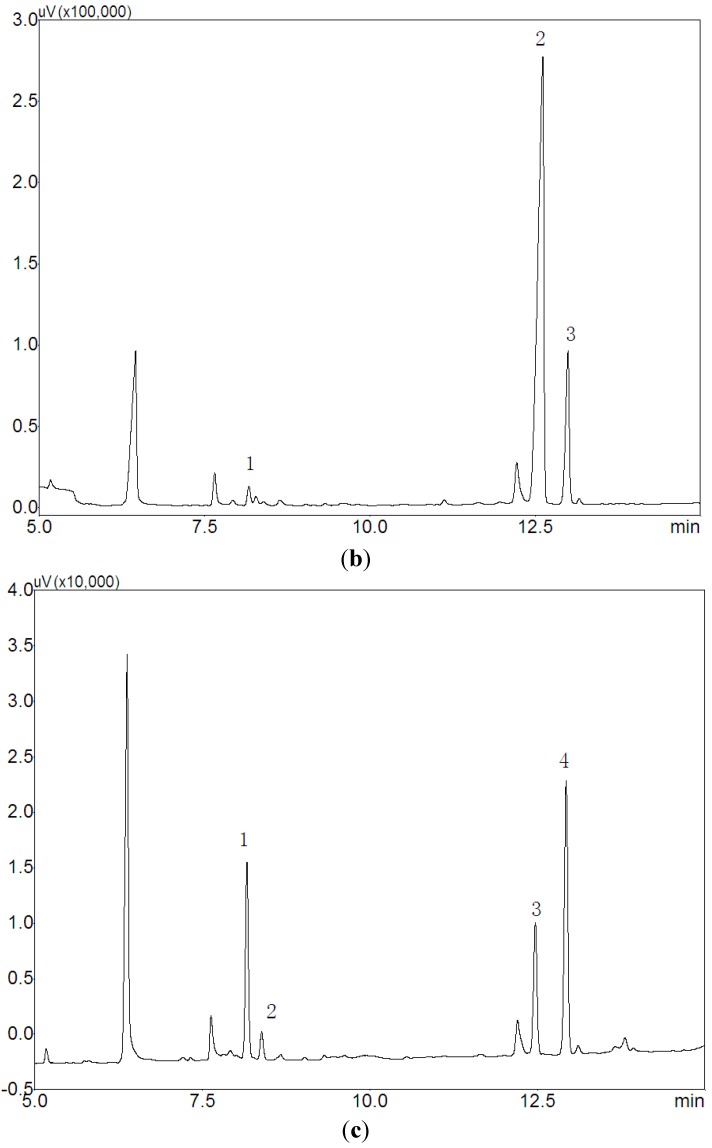

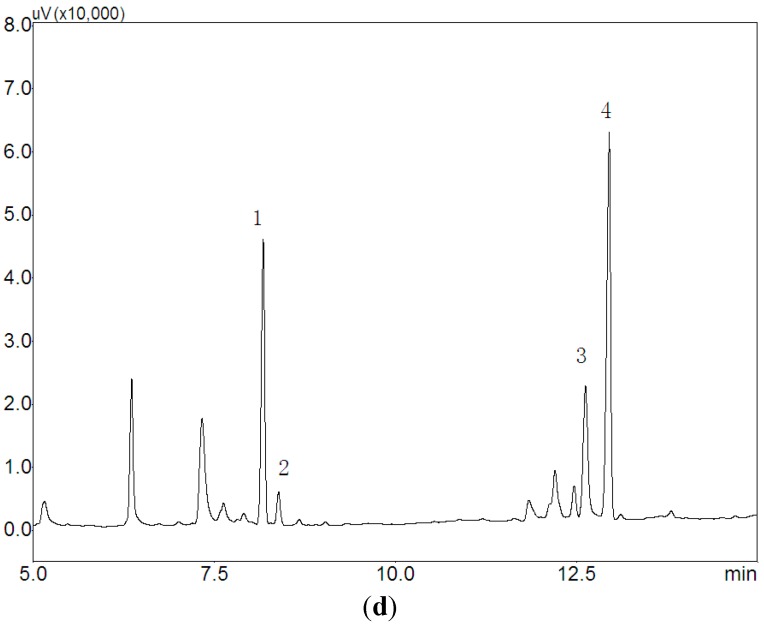
Gas chromatogram of the derivatives of six standard monosaccharide and BSP1A, BSP2A, BSP3B. (**a**) Gas chromatogram of six standard monosaccharides (1, Rhamnose; 2, arabinose; 3, xylose; 4, glucose; 5, mannose; 6, galactose); (**b**) Gas chromatogram of the derivatives of BSP1A (1, arabinose; 2, glucose; 3, galactose); (**c**) Gas chromatogram of the derivatives of BSP2A (1, arabinose; 2, xylose; 3, glucose; 4, galactose); (**d**) Gas chromatogram of the derivatives of BSP3B (1, arabinose; 2, xylose; 3, glucose; 4, galactose).

The six standard monosaccharides were sequenced as follows: rhamnose (1), arabinose (2), xylose (3), glucose (4), mannose (5), and galactose (6) (Figure 3a). BSP1A consisted of arabinose, glucose, and galactose at a molar ratio of 1:40.6:8.7 (Figure 3b). BSP2A and BSP3B contained arabinose, xylose, glucose, and galactose at molar ratios of 6.6:1:5.2:10.4 and 8.5:1:5.1:11.1(Figure 3c,d), respectively.

### 2.4. Carbohydrate-Peptide Linkage Analysis

**Figure 4:**
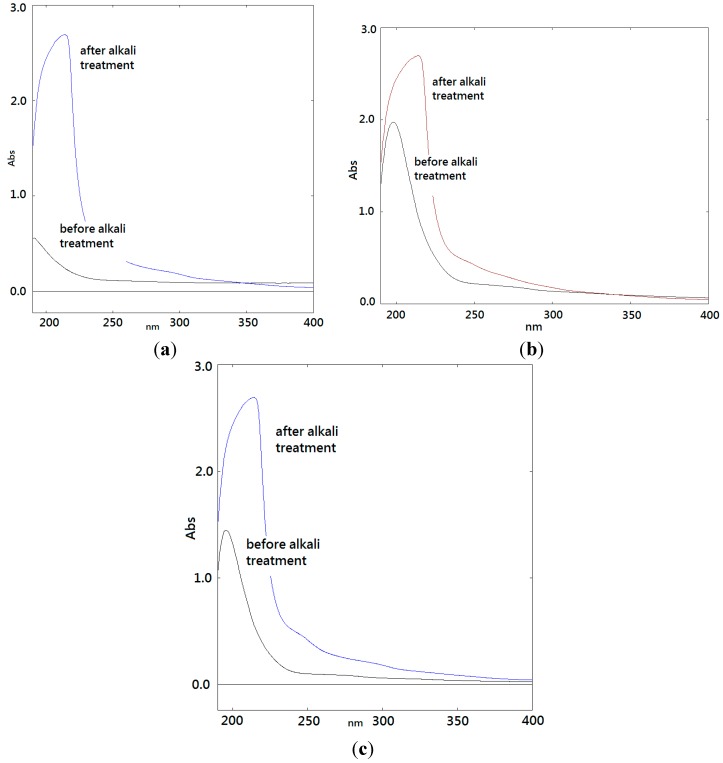
UV spectrum profiles of bamboo shoots polysaccharides and the alkali-treated sample. (**a**) BSP1A; (**b**) BSP2A; (**c**) BSP3B.

After alkaline hydrolysis, the UV scanning spectrum solution absorbance at 240 nm of BSP1A, BSP2A, and BSP3B significantly increased, indicating that these polysaccharides exhibited an *O*-polysaccharide glycosidic bond (Figure 4a–c).

Sodium borohydride (0.1 M, 5 mL) was added to the β-elimination reaction, α-acrylic acid was reduced to alanine, and α-amino crotonic acid was reduced to α-amino butyric acid. A certain relationship existed between the increase of α-alanine and aminobutyric acid and the decrease of serine and threonine in the β-elimination reaction. Up to 0.24 mol alanine increased with the loss of 0.09 serine and 0.22 mol threonine per mole of BSP1A; 0.12 mol alanine increased with the loss of 0.09 serine and 0.07 mol threonine per mole of BSP2A; 0.20 mol alanine increased with the loss of 0.13 serine and 0.15 mol threonine per mole of BSP3B (Table 1 and Table 2). Content changes in the amino acids of the three polysaccharides indicated that BSP1A, BSP2A, and BSP3B exhibited *O*-peptide bond (-*O*-Ser or -*O*-Thr) connections [12].

**Table 1:** Contents of amino acids in BSP1A, BSP2A and BSP3B before and after 0.1 mol/L NaOH treatments.

Amino Acids	BSP1A (mg/g)		BSP2A (mg/g)		BSP3B (mg/g)	
	Before	After	Before	After	Before	After
Asp	1.72	1.47	1.94	1.67	1.54	1.32
Thr	3.53	1.95	2.75	2.24	3.13	2.25
Ser	1.18	0.42	0.98	0.52	1.36	0.78
Glu	1.36	2.12	1.52	1.50	1.15	1.47
Gly	0.71	1.13	0.88	1.12	0.76	0.98
Ala	3.57	5.26	2.56	3.08	2.76	3.48
Cys	1.96	1.53	1.34	1.05	2.96	2.53
Val	1.55	1.26	1.12	1.35	1.35	1.48
Met	–	0.43	–	0.52	–	0.43
Ile	0.52	0.94	0.61	0.86	0.52	0.94
Leu	1.41	1.28	0.20	0.29	1.28	1.02
Typ	–	0.42	0.36	–	0.52	0.22
Phe	0.72	–	0.64	–	0.34	–
Lys	1.27	0.98	1.06	0.82	0.47	0.18
His	3.12	2.13	1.42	1.06	1.94	1.96
Arg	0.53	–	0.42	–	0.57	–
Pro	1.21	1.33	0.92	1.05	0.95	1.23
Hdr	–	–	–	–	–	–
The total content of amino acids	24.36	22.35	18.72	17.13	22.36	20.27
The protein content	23.48	–	17.86	–	21.22	–

**Table 2:** Changes of amino acid residues per mole of BSP1A, BSP2A and BSP3B before and after alkali-β-elimination.

Sample	Amino Acid Residues	Before	After	Difference
BSP1A	Thr	0.30	0.08	0.22
	Ser	0.14	0.05	0.09
	Ala	0.52	0.76	0.24
BSP2A	Thr	0.39	0.32	0.07
	Ser	0.14	0.05	0.09
	Ala	0.61	0.73	0.12
BSP3B	Thr	0.56	0.41	0.15
	Ser	0.31	0.18	0.13
	Ala	0.78	0.98	0.20

### 2.5. FTIR Spectra

**Figure 5:**
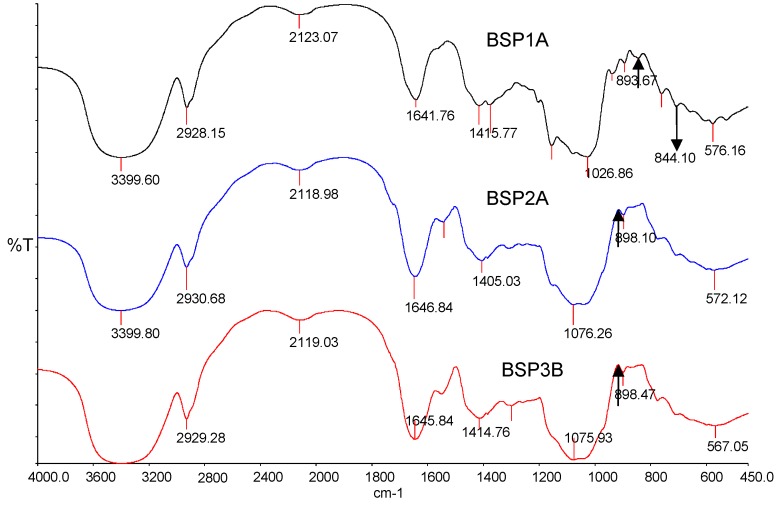
Infrared spectrum of BSP1A, BSP2A and BSP3B (The arrows pointed at the peak absorptions that were about 892 ± 7 cm^−1^).

The FTIR spectra (Figure 5) were used to provide further structural information about BSP1A, BSP2A, and BSP3B. Different absorption bands for FTIR analysis were assigned as previously described in the literature. In 3399.60, 3399.80, 3399.50 and 2928.15, 2930.68, 2929.18 cm^−1^ of the three polysaccharides, the infrared spectra were attributed to the OH stretching vibrations in hydrogen bonds and the C-H stretching vibrations (Figure 5). In 1641.76, 1646.84, 1645.84 and 1415.77, 1405.03, 1414.76 cm^−1^ of the three polysaccharides, the infrared spectra were ascribed to the absorption of the COO^−^ deprotonated carboxylic group [13]. The absorption peaks at 1026.86, 1076.26, and 1075.93 cm^−1^ indicated the presence of a β-pyran linkage. The three polysaccharides’ peak absorptions were about 892 ± 7 cm^−1^, indicating β-glycopyranosidic linkages. Only BSP1A showed an absorption peak at 844.10 cm^−1^, implying that BSP1A exhibited α-configurations. Overall, both BSP2A and BSP3B displayed β-d-pyran-type sugar rings, and the polysaccharide molecules may be connected to the β-type glycosidic bond; however, BSP1A showed both β-d-pyran-type and α-d-pyran-type sugar rings [14].

### 2.6. Triple Helix Structure Analysis

**Figure 6:**
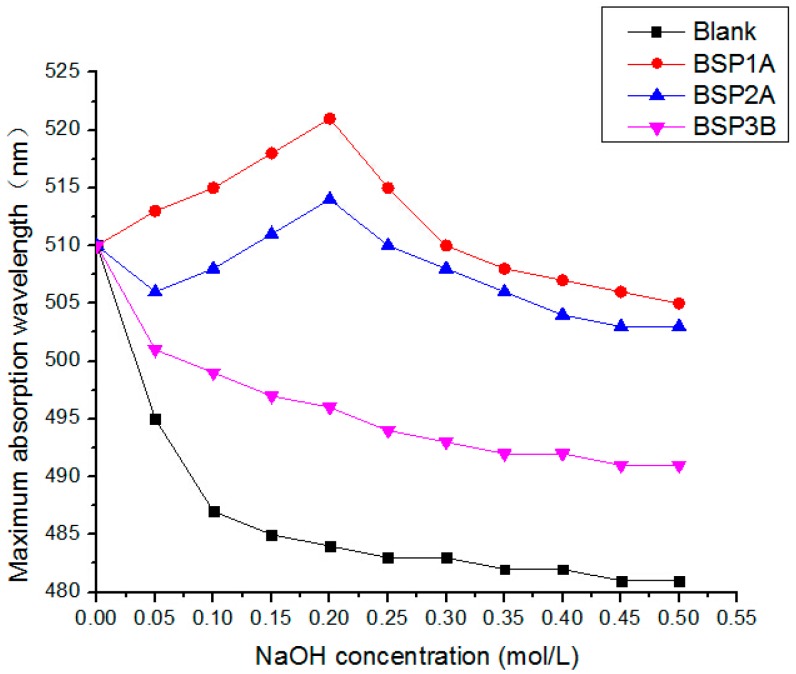
Dependence of the λ max of Congo Red and BSP1A, BSP2A, BSP3B complex on NaOH concentration.

Congo red is an acid dye that can form triple helix structures. The maximum absorption wavelength of the complex with polysaccharides changes compared with Congo red [15]. With increasing concentrations of NaOH, the maximum absorption wavelength of the complex combined with BSP1A and BSP2A first increased then decreased, and the concentration of NaOH in 0.2 mol/L was the largest (Figure 6). The BSP3B maximum absorption wavelength was reduced, and its maximum wavelength significantly decreased slowly compared with Congo red. From 0.2 to 0.5 mol/L, the maximum absorption wavelength decreased continuously probably because the remaining hydrogen bonds of the bamboo shoot (*D. latiflorus*) polysaccharide were destroyed by the large alkaline. Therefore, BSP1A and BSP2A may exhibit a triple helix structure, whereas BSP3B may not present such structure.

### 2.7. NMR Spectroscopy Results

**Figure 7:**
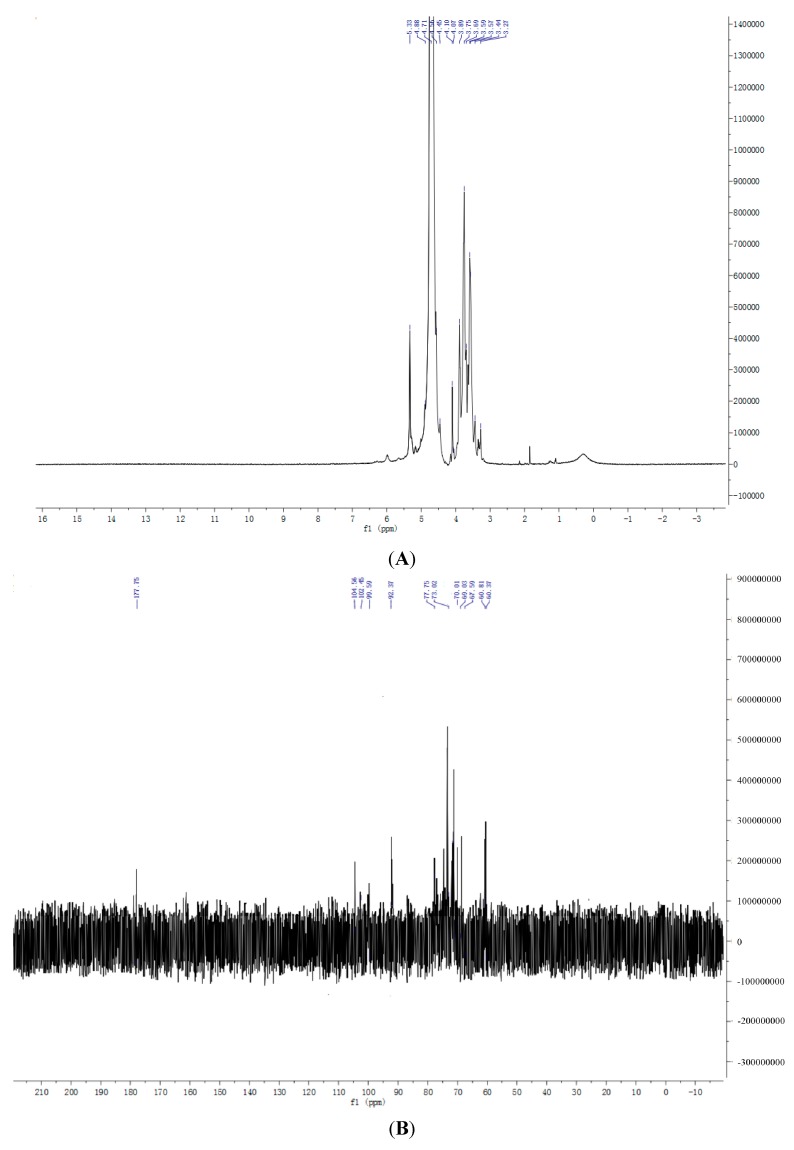

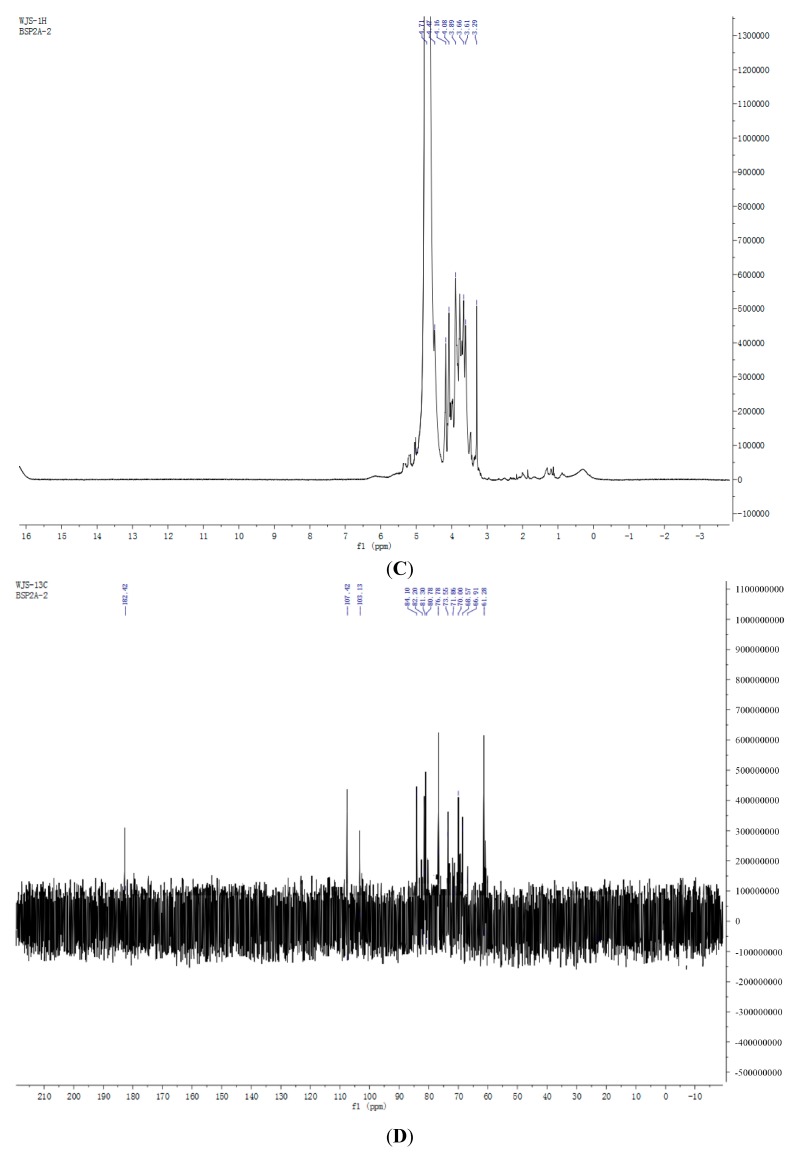

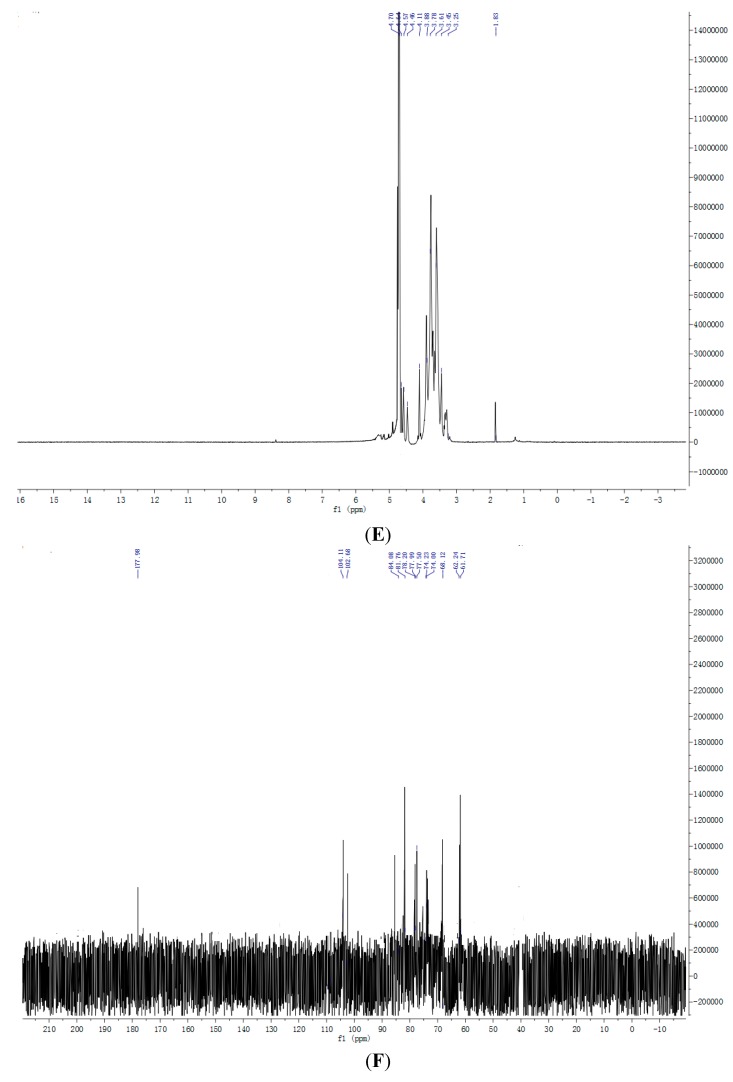
(**A**) ^1^H NMR spectrum of BSP1A; (**B**) ^13^C NMR spectrum of BSP1A; (**C**) ^1^H NMR spectrum of BSP2A; (**D**) ^13^C NMR spectrum of BSP2A; (**E**) ^1^H NMR spectrum of BSP3B; (**F**) ^13^C NMR spectrum of BSP3B.

NMR spectroscopy of ^1^H and ^13^C is an efficient method to analyze the structural features of bamboo shoot polysaccharides. The signals from anomeric protons at δ5.33, δ3.75, and δ3.89 showed that BSP1A demonstrated both α- and β-type configurations (Figure 7A). The anomeric proton signal at δ4.47 revealed that BSP2A was a β-type configuration (Figure 7C). The anomeric proton signals at δ4.57 and δ4.46 indicated that BSP3B was a β-type configuration (Figure 7E). Given that no anomeric proton existed at δ5.40, BSP1A, BSP2A, and BSP3B were pyran-type sugar rings. These findings were consistent with infrared spectrum analysis.

The anomeric region of ^13^C NMR (95–110) showed three main signals; the signal at δ104.34 and δ102.45 showed that BSP1A presented a β-type configuration, and the signal at δ99.59 revealed that BSP1A displayed an α-type configuration (Figure 7B). Calculation based on the peak height was α:β = 13.7:1, indicating that BSP1A mainly existed in the β type. The signal at δ92.37 was attributed to glucose; some other anomeric carbon signals were weak, which may be ascribed to different configurations of galactose and arabianose. No signals existed at δ78–δ85, indicating that no substitution reaction occurred in the C-2, C-3, C-4. Thus, BSP1A did not demonstrate 1→2, 1→3 and 1→4 glycosidic bonds. The anomeric carbon signals at δ67.59, δ69.03, and δ70.01 indicated the existence of 1→6 glycosidic bond. BSP1A monosaccharide composition analysis showed →6)β-Glu(1→ was the main glycosidic linkage of BSP1A, and few galactopyranose and arabinopyranose bonds were connected with the position and configuration, which demanded further research [16].

The anomeric region of ^13^C NMR (95–110) showed two main signals, and the signals at δ107.42 and δ103.13 showed that BSP2A presented a β-type configuration (Figure 7D). Given the signals at δ70 to δ85 that densely overlapped and combined with the monosaccharide composition analysis, BSP2A was considered a heterosaccharide. The signals at δ107.42, δ81.30, δ76.78, δ84.10, and δ68.57 can be assigned to the carboxyl groups C-1, C-2, C-3, C-4, and C-5 of the glycosidic bond of →5)β-Ara(1→ [17]. The signals at δ103.13, δ70.00, δ81.03, δ73.55, δ76.78, and δ68.57 can be assigned to the carboxyl groups C-1, C-2, C-3, C-4, C-5, and C-6 of the glycosidic bond of →3)β-Gal(1→ [18]. The signal was weak in other places; hence, the response signal belonging to the bond was difficult to evaluate. Combined with ^1^H NMR and ^13^C NMR spectra, BSP2A probably exhibited two main glycosidic bonds, →3)β-Gal(1→ and →5)β-Ara(1→, and presented other glycosidic bond connections, which required further exploration.

Compared with ^13^C NMR, ^1^H NMR of BSP3B with BSP2A showed similar anomeric protons (Figure 7F). Similarly, BSP3B presented two main glycosidic bonds, namely, →3)β-Gal(1→ and →3)β-Glu(1→, with other glycosidic bond connections, which required further examination [19].

## 3. Experimental Section

### 3.1. Plant Material

Bamboo shoots (*D. latiflorus*) were purchased from agricultural markets of Beibei, Chongqing, China. The plant material was cut into pieces, oven dried at 55 °C, and then crushed into powder [20].

### 3.2. Chemicals

DEAE-Cellulose-52 and T-dextran series of different standard molecular weights were purchased from Beijing Soledad Science and Technology Co., Ltd. (Beijing, China). Standard monosaccharides were purchased from China Institute for Food and Drug Control (Beijing, China, an agency for the supply of standard substances of the drug substance in mainland China). Sephadex G-50 and Sephadex G-100 were purchased from Pharmacia (Sweden). Other reagents used were of analytical grade and supplied by Kelong Chemical Reagent Factory (Chengdu, China).

### 3.3. Isolation and Purification of Bamboo Shoot Polysaccharides

Raw polysaccharide was extracted from bamboo shoot through hot water extraction methods, after which it was treated by deproteinization, water dialysis, DEAE-52 cellulose, and Sephadex-50 column chromatography grading. The results showed that papain combined with Sevag presented the optimum deproteinizing condition. The bamboo shoot polysaccharides were eluted with water, 0.05, 0.1, and 0.2 and 0.5 mol/L NaCl salt solution by DEAE-Cellulose 52 column grading; finally, the main components were eluted with water by Sephadex-50 grading [21].

### 3.4. Analytical Methods

Carbohydrate content was determined by phenol-sulfuric acid method with glucose as standard [22].

### 3.5. Determination of Molecular Weight

SephadexG-100 was used to fill the column with specification of Φ1.6 × 70 cm. Water was used for balance for 12 h. The elution rate was 0.5 mL/min, and the eluent was distilled water. The blue dextran standards (Dextran T-2000, Pharmacia, Stockholm, Sweden) on the sample volume were 2 mg each. Initially, the blue dextran elution volume was V_0_. Model T-110, T-70, T-40, and T-10 dextran standards were then applied to the column. The eluent was manually collected, with each tube measuring 3 mL. Phenol-sulfuric acid method was used for tracking and detection, and the elution volume Ve was measured on the basis of the absorbance. The standard curve was drafted with Ve/V_0_ as vertical axis and the natural logarithm of the molecular weight (lg*M*r) as abscissa. Under the same elution condition, 2 mg of purified polysaccharide was dissolved and added to the column; each elution volume was measured as Ve', and the molecular weight values were calculated corresponding to the Ve'/V_0_ in conjunction with the standard curve [23,24].

### 3.6. Determination of Monosaccharide Composition

Polysaccharides (4 mg) were hydrolyzed with 2 mol/L trifluoroacetic acid (4 mL) at 100 °C for 4 h. The hydrolyzate was evaporated with a rotary evaporator. The dried hydrolyzed sample was dissolved and incubated in 0.250 mL of 1 M ammonium hydroxide containing 10 mg/mL NaBH_4_ at room temperature for 3 h. After complete removal of borate ions, the sample was acetylated with 0.2 mL of pyridine and 0.2 mL of acetic anhydride overnight at room temperature and then dried under N_2_ stream. A mixture of chloroform and water was added to the sample, followed by vortexing. The organic phase was concentrated under N_2_ stream and analyzed by GC. GC was performed using Shimadzu GC2010 (Shimadzu, Kyoto, Japan), which was equipped with a capillary column of Rtx-5ms (30.0 m × 0.25 mm × 0.25 μm). The temperature program was conducted as follows: 80 °C for 2 min, 210 °C at 6 °C/min, then to 215 °C at 1.0 °C/min, and finally to 240 °C at 5 °C/min for 2 min. N_2_ was used as carrier gas at 0.8 mL/min [25].

### 3.7. Carbohydrate-Peptide Linkage Analysis

Carbohydrate-peptide linkages of BSP1A, BSP2A, and BSP3B were analyzed by β-elimination reaction. Samples (5 mg/mL) were incubated in 0.1 mol/L NaOH containing 1.0 mol/L NaBH_4_ at 40 °C for 12 h and then scanned by UV spectrophotometry from 190 to 400 nm. The obtained data were compared with those of the alkali-untreated samples. After treatment, each of 30 mg amounts of BSP2A, BSP1A, and BSP3B (before and after β-elimination reaction) were detected on automatic amino acid analyzer (Hitachi type L8800, Hitachi, Tokyo, Japan) [26].

### 3.8. Fourier Transform Infrared (FTIR)

Up to 3 mg of dried bamboo shoot polysaccharide was added to 200 mg KBr powder, gently ground in an agate mortar with infrared lamp, and then pressed into KBr tablets. Subsequently, the tablets were scanned from 4000 to 400 cm^−1^ [27,28].

### 3.9. Congo Red Test

In accordance to Satitmanwiwat’s methods with slight modification, 5 mg of each polysaccharide was added to 2 mL of distilled water and 2 mL of Congo red reagent; different volumes of 1 mol/L NaOH solution were then added to obtain a final concentration of 0 mol/L, which was gradually increased to 0.5 mol/L (0, 0.05, 0.1, 0.15, 0.2, 0.25, 0.3, 0.35, 0.4, 0.45 and 0.5 mol/L) [29]. The samples were mixed and scanned in the ultraviolet wavelength range of 400 to 600 nm. The maximum absorption wavelengths of the sample solution under different alkaline conditions were recorded [30].

### 3.10. NMR Spectroscopy

Spin systems in the polysaccharides and their sequential assignments were identified by recording NMR spectra on a 600 MHz Bruker Avance II spectrometer (Bruker, Stockholm, Sweden). Up to 20 mg each of BSP1A, BSP2A, and BSP3B were collected in three NMR tubes and then dissolved in 0.5 mL of D_2_O at room temperature, separately. ^1^H NMR and ^13^C NMR spectra were measured using a superconducting magnetic resonance instrument [21].

## 4. Conclusions

In this study, three polysaccharides, namely, BSP1A, BSP2A, and BSP3B, were isolated from raw *D. latiflorus* bamboo shoot. The monosaccharide composition of BSP1A was different from that of BSP2A and BSP3B. The existence of *O*-glycopeptide bond in BSP1A, BSP2A, and BSP3B was demonstrated by β-elimination reaction. BSP1A, BSP2A, and BSP3B may be connected to the β-type glycosidic bond. BSP1A and BSP2A may exhibit triple helix structures, whereas BSP3B may not. Both BSP2A and BSP3B displayed β-d-pyran-type sugar rings, and the polysaccharide molecules may be connected to the β-type glycosidic bond. However, BSP1A may present both β-d-pyran-type and α-d-pyran-type sugar rings. BSP1A may exhibit β-1,6-Glucan pyran type as the main link, and few 1,6-glycosidic galactose, pyranose, and arabianose bonds were connected; BSP2A mainly demonstrated →5)β-Ara(1→and→3)β-Gal(1→(connection, BSP3B mainly showed →3)β-Glu(1→and →3)β-Gal(1→connection and few other glycosidic bonds, which require further investigation. This finding about the structure of *D. latiflorus* bamboo shoot polysaccharides suggested that such polysaccharides may perform antioxidant and immunological activities [31]. Further studies are necessary to elucidate the antioxidant behavior of these polysaccharides and worth investigating.
